# Diffraction gratings metrology and ray-tracing results for an XUV Raman spectrometer at FLASH

**DOI:** 10.1107/S1600577517013066

**Published:** 2018-01-01

**Authors:** Siarhei Dziarzhytski, Frank Siewert, Andrey Sokolov, Grzegorz Gwalt, Tino Seliger, Michael Rübhausen, Holger Weigelt, Günter Brenner

**Affiliations:** a DESY, Notkestrasse 85, Hamburg 22607, Germany; b Helmholz-Zentrum Berlin für Materialien und Energie, Albert-Einstein-Strasse 15, Berlin 12489, Germany; c University of Hamburg, Notkestrasse 85, Hamburg 22607, Germany; d Center for Free-Elektron Laser Science, Notkestrasse 85, Hamburg 22607, Germany

**Keywords:** X-ray optics, XUV Raman spectrometer, metrology for synchrotron optics, NOM, reflectometry, ray tracing

## Abstract

The extreme-ultraviolet double-stage imaging Raman spectrometer is a permanent experimental endstation at the plane-grating monochromator beamline branch PG1 at FLASH at DESY in Hamburg, Germany. This unique instrument covers the photon energy range from 20 to 200 eV with high energy resolution of about 2 to 20 meV (design values) featuring an efficient elastic line suppression as well as effective stray light rejection. Such a design enables studies of low-energy excitations like, for example, phonons in solids close to the vicinity of the elastic line. The Raman spectrometer effectively operates with four reflective off-axial parabolic mirrors and two plane-grating units. The optics quality and their precise alignment are crucial to guarantee best performance of the instrument. Here, results on a comprehensive investigation of the quality of the spectrometer diffraction gratings are presented. The gratings have been characterized by *ex situ* metrology at the BESSY-II Optics Laboratory, employing slope measuring deflectometry and interferometry as well as atomic force microscopy studies. The efficiency of these key optical elements has been measured at the at-wavelength metrology laboratory using the reflectometer at the BESSY-II Optics beamline. Also, the metrology results are discussed with respect to the expected resolving power of the instrument by including them in ray-tracing studies of the instrument.

## Abstract 2

Investigations of the diffraction gratings for the high-resolution XUV Raman spectrometer at FLASH *via* both *ex situ* and *in situ* metrology and ray tracing.

## 1. Introduction   

**Figure 1:**
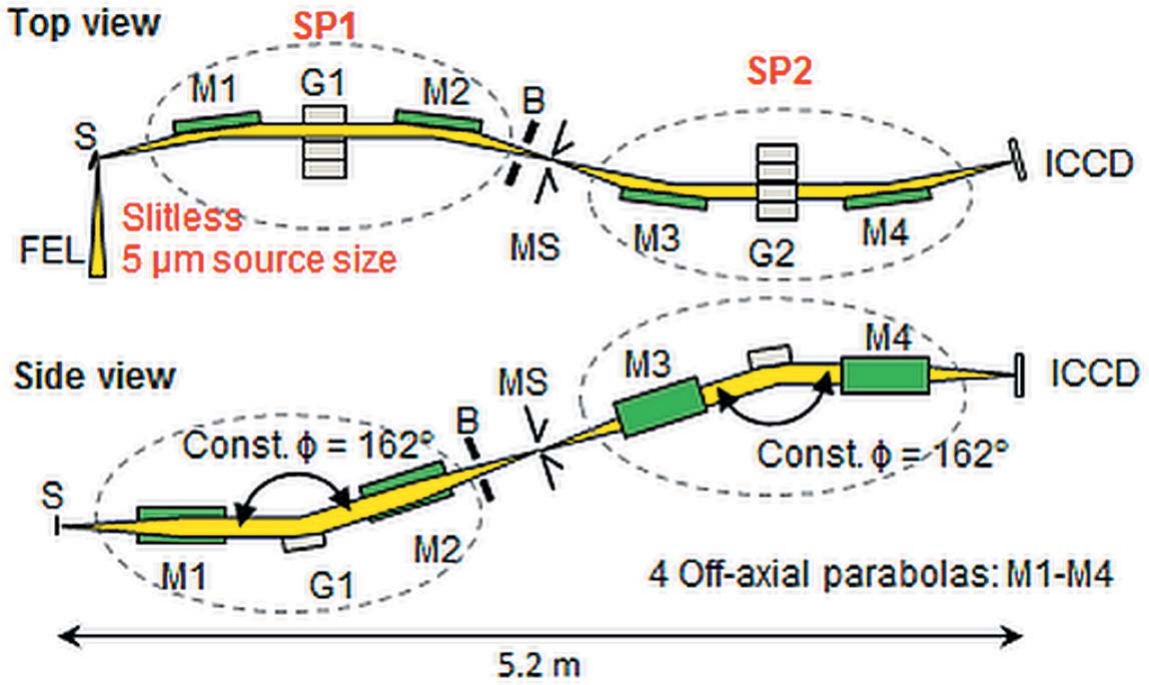
Optical scheme of the XUV double-stage Raman spectrometer at FLASH with free-electron laser beam FEL, spectrometer stages SP1 and SP2, off-axial parabolic mirrors M1–M4, grating units G1 and G2, baffles B, middle slit unit MS, and ICCD as detector. The SP1 stage collects elastically and inelastically scattered photons from the sample S and focuses them after dispersion in the vertical plane onto the middle slit MS, which works as a source for the second spectrometer stage SP2. At SP2 the signal from the sample is further spectrally resolved and recorded by an in-vacuum intensified ICCD camera. The spectrometer works with a constant deviation angle of 162° (Rübhausen *et al.*, 2004 ▸).

The plane-grating monochromator beamline PG1 (Gerasimova *et al.*, 2011 ▸; Dziarzhytski *et al.*, 2016 ▸) at the soft X-ray/extreme-ultraviolet (XUV) free-electron laser FLASH in Hamburg (Ackermann *et al.*, 2007 ▸; Tiedtke *et al.*, 2009 ▸) is permanently equipped with a unique high-resolution XUV double-stage Raman spectrometer, dedicated to (resonant) inelastic soft X-ray scattering (IXS) experiments in the spectral region from 20 to 200 eV (Rübhausen *et al.*, 2004 ▸; Rusydi *et al.*, 2014 ▸). The optical design of the spectrometer is based on a confocal additive coupling of two high-resolution monochromators (SP1 and SP2) mediated by a middle slit (MS) (see Fig. 1 ▸).

Each monochromator is equipped with two off-axis parabolic mirrors and a plane grating.^1^ The spectrometer has no entrance slit and disperses along the vertical direction, thus the vertical size of the focal spot produced by the PG1 beamline Kirkpatrick–Baez (KB) refocusing optics on the sample (Dziarzhytski *et al.*, 2016 ▸; Siewert *et al.*, 2010 ▸) together with the resolution of the primary monochromator PG1 defines the resolution of the first spectrometer stage to a large extent. The spectrometer has a designed spectral resolution of 2 to 20 meV.

Such spectral resolution puts high demands on all optical elements in terms of figure and surface quality. In general, a slope error of the plane grating leads to a reduced spectral resolution. However, slope errors of the order of 0.05 arcsec r.m.s. are nowadays achievable. Such a small slope error does not affect the resolving power significantly. Of crucial importance are the slope errors of the parabolic mirrors. The reflection of the mirrors is perpendicular to the dispersion plane and the resolution is proportional to the sagittal slope error multiplied by the ‘forgiveness factor’ 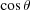, where θ = 7° is the incidence angle of the mirror (Rübhausen *et al.*, 2004 ▸). Off-axis parabolic mirrors of the spectrometer have a sagittal slope error below 1 arcsec. Such values of the optical quality parameters were chosen during the design phase of the spectrometer and pursuit in fabricating to minimize unwanted specular deflections of the rays from their ideal path resulting in reduction of the spectrometer resolution. Also, these values represent the technical limits of parabola production at that time and have been chosen in the closed discussion with the manufacture. Extremely precise metrology instruments are mandatory to characterize high-quality optical elements of the beamline and spectrometer operating in the soft X-ray/VUV spectral range. The Nanometer Optical Component Measuring Machine (NOM) and the atomic force microscope (AFM) at the BESSY-II Optics laboratory (Siewert *et al.*, 2014 ▸) were used in combination with at-wavelength metrology at the BESSY-II Optics beamline (Schäfers *et al.*, 2016 ▸; Sokolov *et al.*, 2016 ▸) of the Helmholtz Zentrum Berlin to characterize the diffraction gratings of the Raman spectrometer. The gratings were produced by Carl Zeiss Optronics GmbH and first tested in 2008. Since then they have been partly used in operation/commissioning but also kept in storage under air pressure for a considerable amount of time. In order to exclude possible performance issues due to coating delamination or other unwanted degradation effects the coating quality and the overall grating efficiency have been thoroughly re-characterized. Parabolic mirrors have not been re-measured in the present work as they were kept in the spectrometer since their production and characterization at Carl Zeiss Optronics GmbH in 2006.

The obtained results from metrology demonstrated some efficiency degradation and deviations from the optics specifications, as will be discussed in the following. These findings were implemented into ray-tracing package *SHADOW* (Cerrina & Sanches del Rio, 2010 ▸) to quantify their influence on the performance of the XUV Raman spectrometer (§3).

## 2. Gratings metrology   

The spectrometer gratings are plane and blazed to yield maximum efficiency in first order. They are mechanically ruled, ion etched and coated with diamond-like carbon (DLC). The substrate material is Zerodur and the coating thickness is 45 nm. All gratings have been characterized *ex situ*. The at-wavelength efficiency of the gratings G1-3 and G2-3^2^ were also measured with the reflectometer at the BESSY-II Optics beamline.

### 2.1. *Ex situ* metrology   

**Figure 2:**
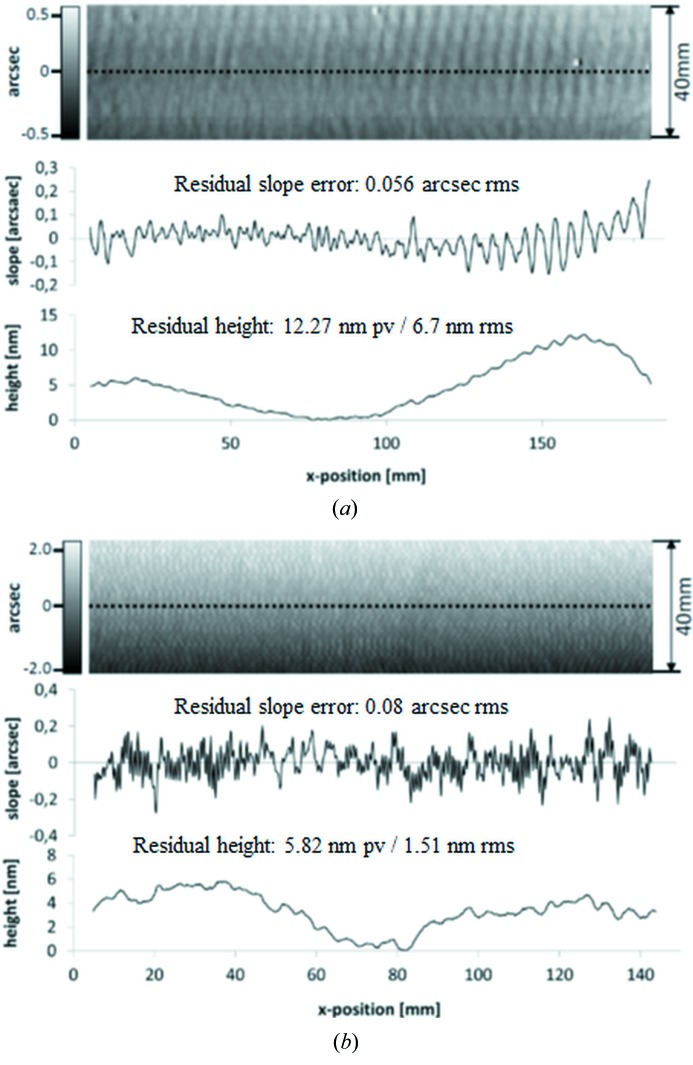
Result of slope measurements on grating G1-3 (*a*) and G2-3 (*b*): slope map in the meridional direction (upper section), profile of the residual slope along the central line (middle), and profile of the residual height (bottom).

**Figure 3:**
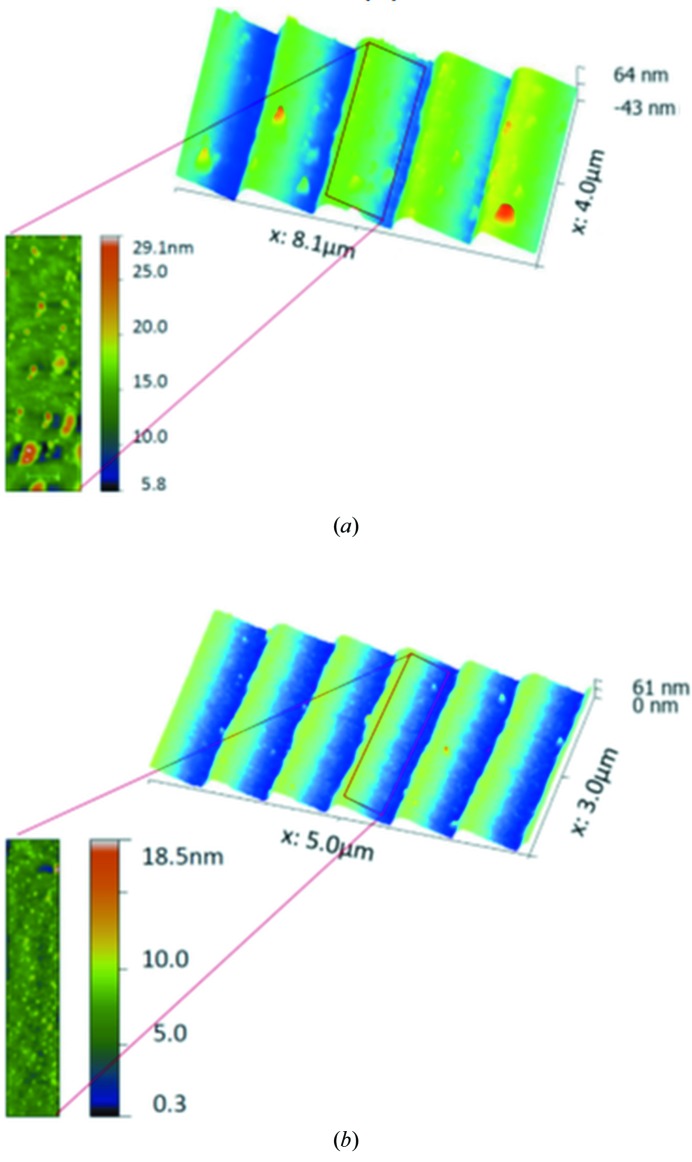
Results of an AFM measurement on grating G1-3 (*a*) and grating G2-3 (*b*), showing the blaze profile and the state of the micro-roughness on the grooves.

Spectrometer gratings have been characterized *ex situ* by means of the BESSY-NOM (Siewert *et al.*, 2004 ▸), regarding topography in terms of slope, and curvature in terms of the substrate meridional radius. The sagittal slope error was not measured due to the forgiveness factor assumption for the application of the grating. AFM measurements were made to characterize the groove profile of the gratings regarding blaze profile, groove density and micro-roughness on the grooves. The instrument applied is a NaniteAFM (SPM S200) by Nanosurf. While the NOM measurements allow the spatial frequency range from 1.2 mm up to aperture length to be verified (Siewert *et al.*, 2016 ▸), the AFM gives a view on the nano-topology of the grating with a spatial resolution in the range from ≤10 nm up to a few µm, depending on the tip radius and the field of view applied for such measurements (Breil *et al.*, 2002 ▸). The spatial frequency range covered by the slope error has an impact on the effects of classical aberration. The higher spatial frequency range as measured by means of the AFM has an impact on the efficiency (*e.g.* losing flux because of scattering) and spectral purity provided by the grating. Fig. 2 ▸ shows the results of the slope measurements for the gratings G1-3 and G2-3. Fig. 3 ▸ shows the state of the groove profile and micro-roughness on the grooves as measured by using an AFM.

Table 1 ▸ shows the results of the measurements in detail. The measurements reveal a compliance with the specification for most of the parameters like the slope error, radius of curvature, groove density and blaze angle. The AFM measurements have shown high values for the micro-roughness on the grooves of 1–6 nm r.m.s. for grating G1-3. This is probably because of aging effects during the years of storage. The micro-roughness on grating G2-3 is 0.80–1.61 nm r.m.s. slightly better compared with grating G1-3. However, the micro-roughness is out of specification for both gratings.

### 2.2. *In situ* metrology   

**Figure 4:**
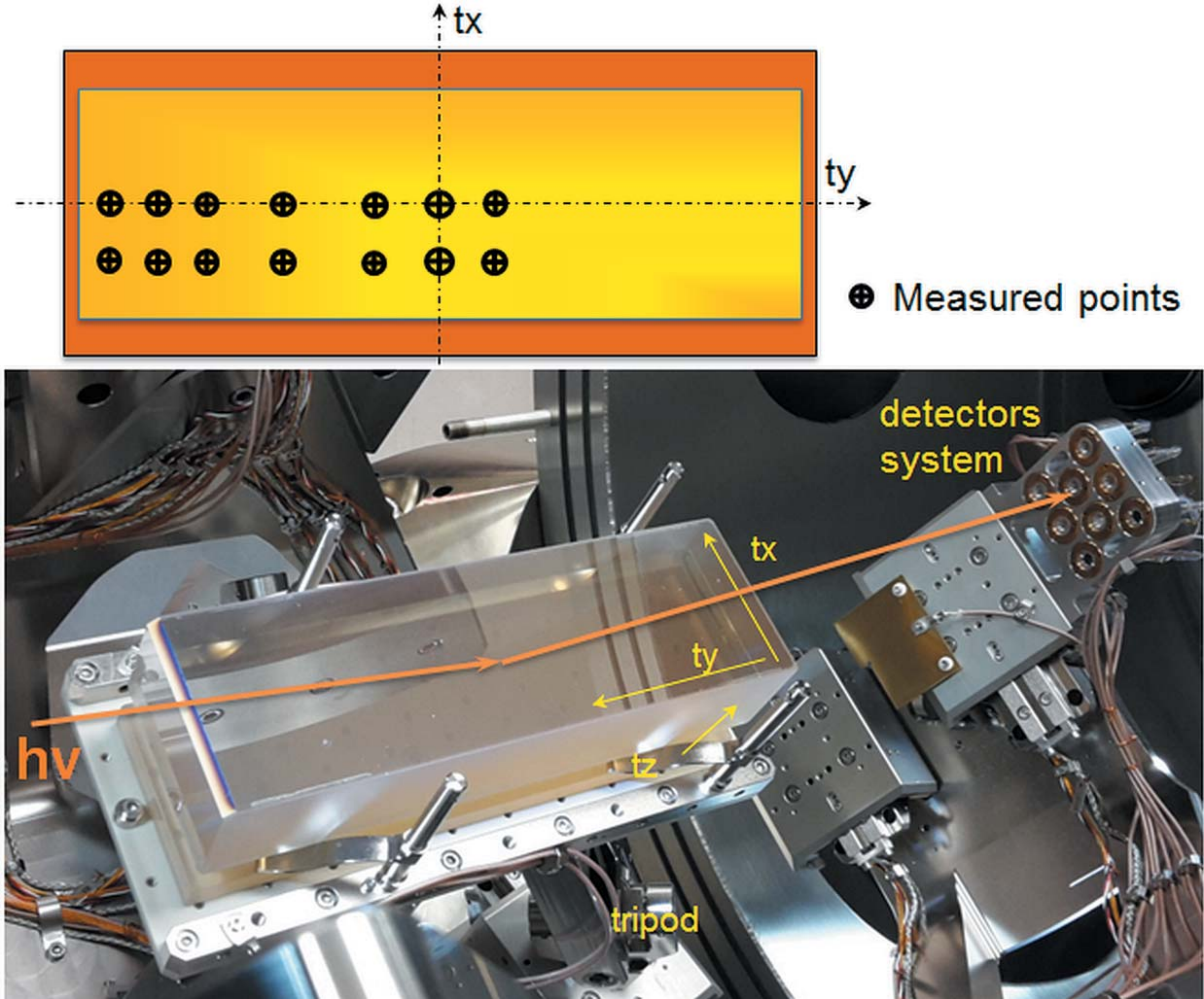
Grating installed at the reflectometer. Beam propagation direction, orientation notation and measured points on the grating are shown.

Diffraction efficiencies of the Raman spectrometer gratings G1-3 and G2-3 have been measured with the reflectometer at the BESSY-II Optics beamline. A standard beam focus size *a*_*x*_ (along the grating grooves) × *a*_*y*_ (across the grooves) of 0.2 mm × 0.36 mm was used, which results in a footprint size of *a*_*x*_ × *a*_*y*_/sin(θ) (where θ is the grazing incidence angle) on the grating. Since the measured area is rather small compared with the total grating working aperture, measurements at different points on gratings have been carried out in order to test a larger grating area (see Fig. 4 ▸).

**Figure 5:**
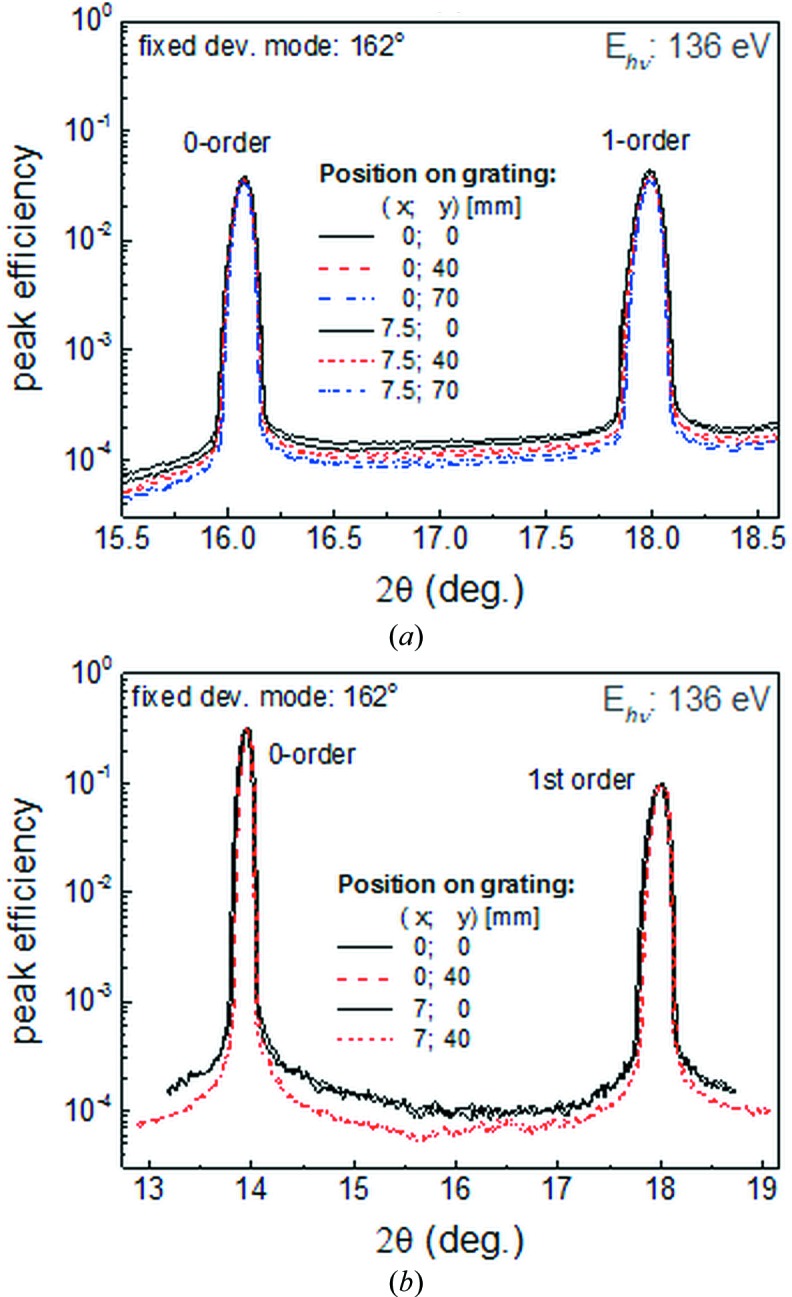
Measured dispersion pattern for grating G1-3 (*a*) and G2-3 (*b*) in the range of the zeroth and first diffraction orders at fixed photon energy of 136 eV and constant deviation mount of 162°. No ghost peaks were observed. The positions on the grating are counted from the center (0, 0), *X* along the grating groove and *Y* along the incoming photon beam axis.

Fig. 5 ▸ presents dispersion scans at fixed photon energy of 136 eV at different positions on both gratings under investigation. For both gratings only a small difference of the low-level background signal was observed. The area between the zeroth- and first-order peaks does not reveal any abnormal structure like a ghost peak or peak shape distortion, which could be related to grating structure defects. In general, both gratings tested exhibit an efficiency variation of only ∼0.1% across the measured points.

**Figure 6:**
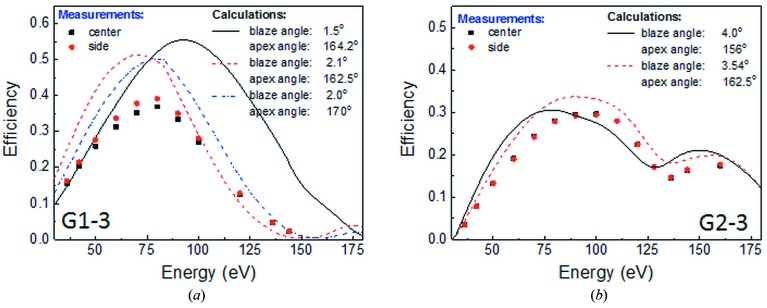
Calculated (lines) and measured (points) efficiencies for grating G1-3 (*a*) and G2-3 (*b*). Calculations were carried out using the *REFLEC* code (Schäfers & Krumrey, 1996 ▸) included in the *RAY* package (Schäfers, 1996 ▸) using grating parameters taken from the specifications (black line) and in the present work (blue and red dashed lines).

However, the measured efficiency energy dependence does not match very well with the efficiency calculations based on grating parameters obtained from the *ex situ* metrologies carried out after the manufacturing and from the present work (see Fig. 6 ▸).

The deviations between the efficiency values measured and calculated are on average in the limits of 10–15% for grating G1-3 and 2–5% for grating G2-3. In the case of grating G2-3 one can see a deviation in the shape of the measured curve compared with the calculated one. This points to possible structural deviation in the grating profile, which is not described by the model used. Overall, in spite of the found efficiency deviations, the investigated gratings show an acceptable performance. The design and the measurements results for gratings G1-3 and G2-3 are compiled in Table 1 ▸.

## 3. Ray tracing   

The spectrometer is designed for high-resolution IXS experiments in the XUV spectral region, thus high-quality optical elements are mandatory to meet the designed performance specifications. Generally, figure and slope error imperfections manifest themselves in specular deflections of the rays from their ideal path, which thus results in focal spot broadening and a possible reduction of the spectrometer resolution. Furthermore, surface roughness (random irregularities in microscopic scale) can lead to a blurring of the image and a loss of contrast at the focus due to wide-angle scattering of the photon rays. The effects of such imperfections on the spectrometer performance are analyzed here.

The measured r.m.s. slope error values were used in the pre-processor ‘WAVINESS’ to simulate maps of the slope errors of the gratings. We also applied measured slope error profiles to the grating surfaces in the ray tracing and compared results of both approaches. The source for the ray tracing has two spectral lines in the vicinity of the blazed energy of the gratings and a rectangular shape with spatial dimensions of 5 µm × 20 µm (V × H) and uniform divergence of 37 mrad × 82 mrad (V × H). Such parameters reflect a realistic experimental PG1 focal size formed by the KB optics on the sample of the Raman spectrometer (Dziarzhytski *et al.*, 2016 ▸).

**Figure 7:**
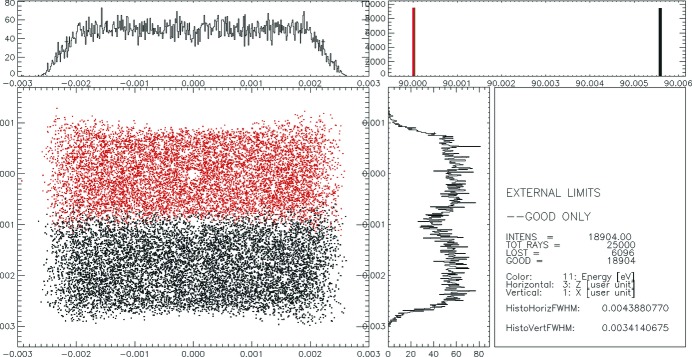
Spectrometer focal spot which accounts for 5.6 meV resolution at a photon energy of 90 eV. Gratings G1-3 and G2-3 were used. No slope errors were applied.

Fig. 7 ▸ demonstrates a spectrometer resolution of 5.6 meV at a photon energy of 90 eV in the ideal case when no slope errors are taken into account.

**Figure 8:**
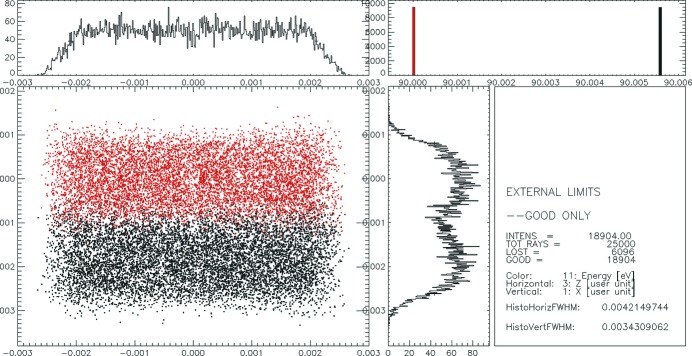
Spectrometer focal spot which accounts for 5.6 meV resolution at a photon energy of 90 eV. The meridional slope error is 0.056 arcsec r.m.s. for grating G1-3 and 0.08 arcsec r.m.s. for G2-3. The sagittal slope error is 0.1 arcsec r.m.s. for both gratings. Meshed slope error surfaces were simulated by *SHADOW* (Cerrina & Sanches del Rio, 2010 ▸).

First, slope errors of 0.056 and 0.08 arcsec r.m.s. in the meridional direction for gratings G1-3 and G2-3, respectively, and 0.1 arcsec r.m.s. in the sagittal directions for both gratings measured by the NOM instrument were used in the pre-processor ‘WAVINESS’ to create maps of slope errors for the gratings. These maps were applied to the surface of the gratings to calculate the focal spot size and also to estimate the resolution of the spectrometer (see Fig. 8 ▸).

**Figure 9:**
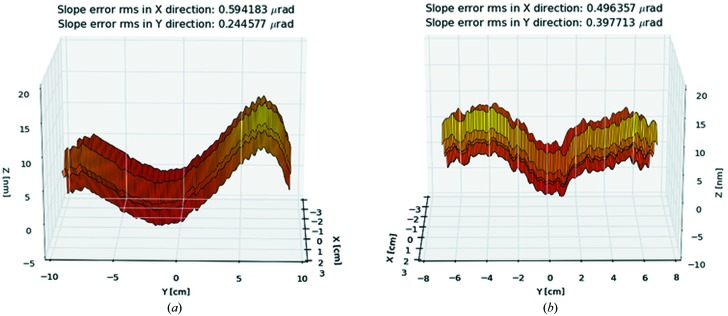
Residual height profile of the gratings G1-3 (*a*) and G2-3 (*b*) reconstructed from the measured one-dimensional profiles.

**Figure 10:**
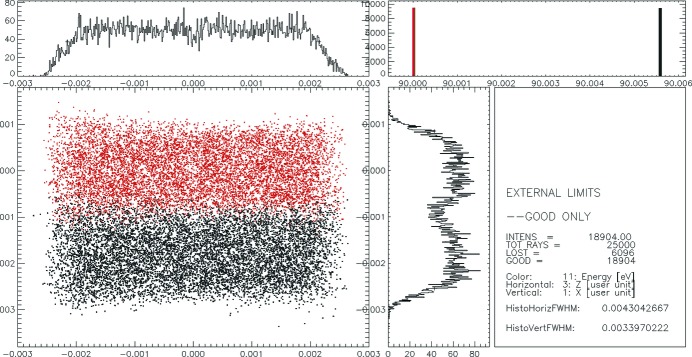
Spectrometer focal spot which accounts for 5.6 meV resolution at a photon energy of 90 eV. Surface splines which are shown in Fig. 9 ▸ are applied.

As one can see from a comparison of Figs. 7 ▸ and 8 the resolution of the spectrometer of 5.6 meV is not affected by the application of the calculated slope errors to the gratings. The vertical focus size is about 35 µm full width at half-maximum (FWHM) in both the ideal case and when slope errors are applied. Furthermore, the three-dimensional maps of the surface error generated out of the one-dimensional profiles measured by the NOM instrument were introduced into the simulations using the ‘PRESURFACE’ pre-processor. Examples of the surface spline for gratings G1-3 and G2-3 are shown in Fig. 9 ▸. The slope error in the *X* direction (along the grooves) was generated for a slope error of 0.1 arcsec r.m.s. for both gratings G1-3 and G2-3. The ray-tracing results with measured surfaces for gratings G1-3 and G2-3 are shown in Fig. 10 ▸.

The resolution of the spectrometer is also not reduced due to the applied measured slope error profiles. The vertical focal size is roughly 36 µm FWHM *versus* 35 µm in the ideal case. The horizontal size of the focal spot is not affected and remains about 43 µm FWHM. In general, the effect of the slope error of the diffraction gratings on the spectrometer resolution is much weaker compared with that from the focusing elements, namely the four (M1–M4) off-axial parabolic mirrors used in the spectrometer. The mirrors’ parameters are summarized in Table 2 ▸.^3^

The spectrometer resolution reduces from 5.6 meV to 10 meV at 90 eV photon energy if the slope errors of the off axial parabolas are taken into account.

**Figure 11:**
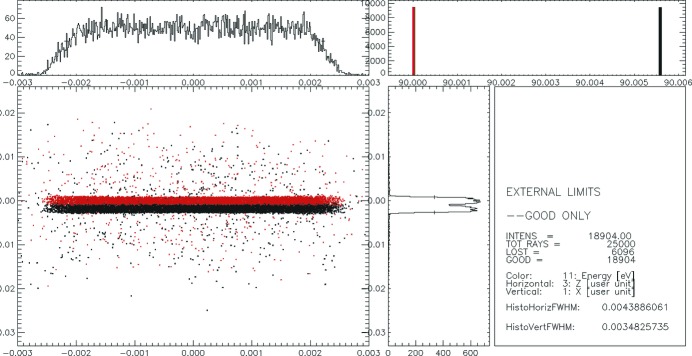
Image of the focal spot after applying surface roughness to the spectrometer gratings.

The effect of the measured micro-roughness was also estimated. The power spectral density function (PSD) was created by means of the JNTPSCALC tool in *SHADOW*. A Gaussian correlation function was chosen with correlation length of 2 cm^−1^ for grating G1-3 and 5 cm^−1^ for grating G2-3. RMS values of the surface roughness of 60 and 16 Å in the *X* and *Y* directions were taken for gratings G1-3 and G2-3, respectively. The results are shown in Fig. 11 ▸.

The measured micro-roughness is out of specification for both gratings which leads to a blurring effect of the image and a loss of contrast at the focus as well as less efficient suppression of unwanted scattered light. However, the signal-to-background ratio in this case is still high enough to use these gratings in the spectrometer.

## 4. Conclusion   

Our optics metrology and ray tracing have clearly demonstrated that, although the spectrometer diffraction gratings had experienced some degradation in efficiency and roughness, no strong negative effect on the slope error and micro-roughness due to possible suspected coating delamination and other processes has been revealed. The gratings provide reasonable efficiency and can be further used for the high-resolution RIXS experiments at the XUV Raman spectrometer at the PG1 beamline at FLASH.

## Figures and Tables

**Table 1:** Diffraction grating specifications and measurements results

Grating	G1-3		G2-3	
Parameters	Specified	Measured	Specified	Measured
Groove density (mm^−1^)	576	576	1224	1224
Spectral range (eV)	36–144		36–144	
Coating	DLC (45 µm thickness)		DLC (45 µm thickness)	
Blaze angle (°)	1.5 ± 0.2	2.09 ± 0.2	4 ± 0.4	3.54 ± 0.1
Blaze energy (eV)	90	80	90	90
Efficiency (%)	45 (at 90 eV)	35 (at 90 eV)	30	29
	Maximum: 39 (at 80 eV)			
Ruling dimensions, L × W (mm)	175 × 54		142 × 54	
Radius (km)	>20	∼146	>20	>500
Slope error r.m.s. (arcsec)	Meridional <0.05	Meridional ∼0.056	Meridional <0.05	Meridional ∼0.08
	Sagittal <0.1		Sagittal <0.1	
Micro-roughness r.m.s. on the groove (nm)	<1	∼1–6	<1	0.8–1.61

**Table 2:** Off-axial parabolic mirrors specified and measured parameters

Mirrors	M1		M2–M4	
Parameters	Specified	Measured	Specified	Measured
Focal length (mm)	550	550	1200	1200
Dimensions, L × W (mm)	390 × 20		397.5 × 45	
Slope error r.m.s. (arcsec)	Meridional <3	Meridional <0.75	Meridional <1	Meridional <0.96
	Sagittal <1	Sagittal <0.96	Sagittal <1	Sagittal ∼1
Micro-roughness r.m.s (nm)	<0.5	0.38–0.5	<0.5	0.85

## Footnotes

1 In fact, each monochromator stage has a set of four gratings to cover the full spectral range; however, only one grating is used in each stage at a time.

2 Grating G*X*-*Y* corresponds to grating *Y* (*Y* = 1–4) at the monochromator stage *X* (*X* = 1, 2).

3 Mirrors were measured by Carl Zeiss Optronics GmbH in 2006. Data are taken from the production and control report.
